# Development of a Prediction Rule for Estimating Postoperative Pulmonary Complications

**DOI:** 10.1371/journal.pone.0113656

**Published:** 2014-12-01

**Authors:** Byeong-Ho Jeong, Beomsu Shin, Jung Seop Eom, Hongseok Yoo, Wonjun Song, Sangbin Han, Kyung Jong Lee, Kyeongman Jeon, Sang-Won Um, Won-Jung Koh, Gee Young Suh, Man Pyo Chung, Hojoong Kim, O. Jung Kwon, Sookyoung Woo, Hye Yun Park

**Affiliations:** 1 Division of Pulmonary and Critical Care Medicine, Department of Medicine, Samsung Medical Center, Sungkyunkwan University School of Medicine, Gangnam-gu, Seoul, Republic of Korea; 2 Department of Internal Medicine, Pusan National University College of Medicine, Pusan, Republic of Korea; 3 Department of Anesthesiology and Pain Medicine, Samsung Medical Center, Sungkyunkwan University School of Medicine, Gangnam-gu, Seoul, Republic of Korea; 4 Biostatistics Team, Samsung Biomedical Research Institute, Gangnam-gu, Seoul, Republic of Korea; University of Tübingen, Germany

## Abstract

Patient- and procedure-related factors associated with postoperative pulmonary complications (PPCs) have changed over the last decade. Therefore, we sought to identify independent risk factors of PPCs and to develop a clinically applicable scoring system. We retrospectively analyzed clinical data from 2,059 patients who received preoperative evaluations from respiratory physicians between June 2011 and October 2012. A new scoring system for estimating PPCs was developed using beta coefficients of the final multiple regression models. Of the 2,059 patients studied, 140 (6.8%) had PPCs. A multiple logistic regression model revealed seven independent risk factors (with scores in parentheses): age ≥70 years (2 points), current smoker (1 point), the presence of airflow limitation (1 point), American Society of Anesthesiologists class ≥2 (1 point), serum albumin <4 g/dL (1 point), emergency surgery (2 points), and non-laparoscopic abdominal/cardiac/aortic aneurysm repair surgery (4 points). The area under the curve was 0.79 (95% CI, 0.75–0.83) with the newly developed model. The new risk stratification including laparoscopic surgery has a good discriminative ability for estimating PPCs in our study cohort. Further research is needed to validate this new prediction rule.

## Introduction

Postoperative pulmonary complications (PPCs) are one of the most common perioperative adverse events in patients undergoing surgery and contribute to significant increases in morbidity, mortality, and length of postoperative hospital stay [1]. Although the incidence of PPCs (2–19%) is at least as common as cardiac complications (2.5%) following non-cardiac surgery [2]–[4], the protocol on perioperative pulmonary assessment is much less robust than that for cardiovascular risk assessment.

Based on the American College of Physicians (ACP) guidelines for preventing PPCs in patients undergoing non-cardiothoracic surgery [5], [6], two main categories of risks are associated with the development of PPCs: patient-related and procedure-related risk factors. Despite these established factors, there are still various variables that should be evaluated perioperatively. Moreover, the patient- and procedure-related factors associated with PPCs have changed over the last decade. For example, progressive developments in anesthetic techniques and minimally invasive surgery have contributed to a reduction in PPCs, and improved nutritive conditions of patients have influenced the age of patients who were previously considered to be at unacceptably high surgical risk. With current trends, respiratory physicians are increasingly asked to evaluate PPCs risk, particularly in patients with any problems of the respiratory system or advanced age. Therefore, our goals were to identify independent risk factors of PPCs in the current clinical setting and to develop a clinically applicable scoring system.

## Methods

### Design

This retrospective, single-center, observational study with prospectively collected data was conducted at Samsung Medical Center (a 1960-bed, university-affiliated, tertiary referral hospital in Seoul, South Korea) between June 2011 and October 2012. The study was approved by the Institutional Review Board of Samsung Medical Center to review and publish information obtained from patient records. Informed consent was waived because of the retrospective nature of the study and patient data was deidentified prior to data access and analysis.

### Patients and data collection

Over the study period, all consecutive patients consulting respiratory physicians to evaluate the risk of PPCs before surgery were included, and the PPC-related variables of each patient were registered prospectively. The patients were not included in this study with 1) aged younger than 18 years; 2) pulmonary related surgery; and 3) obstetric surgery or any procedure during pregnancy and the patients with organ transplantation were excluded.

Data collection was performed prospectively by respiratory physicians at the time of consultation about the risk of PPCs. The following information was collected: data related to the patient (age, gender, body mass index (BMI), smoking status, alcohol habits, airflow limitation, comorbidities such as congestive heart failure (CHF), mental status, American Society of Anesthesiologists (ASA) physical status classification, serum albumin, serum hemoglobin, and chest radiograph findings) and the surgical operation (type of anesthesia, elective or emergency surgery, and surgical site and specialty), which are identified in the ACP guidelines as PPC-related variables in patients undergoing non-cardiothoracic surgery. The presence of airflow limitation was defined as the ratio of forced expiratory volume in 1 s to forced vital capacity (FEV_1/_FVC) <0.7 and FEV_1_ <80% of predicted value.

The main outcome, PPCs, consisted of in-hospital postoperative events related to the respiratory system, such as respiratory infection, respiratory failure, pleural effusion, atelectasis, pneumothorax, and bronchospasm within the first 7 postoperative days. The details of the definitions of PPCs have been published elsewhere [7].

### Perioperative prevention strategy

During the perioperative period, a lung-expansion maneuver with incentive spirometry was performed in all patients who underwent general anesthesia surgery [8] and deep inspiration, active coughing, and sputum expectoration were encouraged after surgery. Smokers were recommended to quit smoking for at least 2 weeks before surgery. Patients with asthma were treated according to the asthma guidelines [9].

### Statistical analysis

Data are presented as means ± standard deviation or medians (interquartile ranges) for continuous variables and as numbers (percentages) for categorical variables. Some continuous variables, such as age, ASA physical status, and serum albumin, were coded as dummy variables using cut-off points based on the highest Youden's index ([sensitivity + specificity] – 1) [8] for predicting PPCs and the other continuous variables (BMI and serum hemoglobin) were coded as dummy variables using published threshold values (*see* Table S1) [7], [10]. Based on the odds ratio and p-value of the initial multivariable logistic regression, the surgical specialty in Table 1 was re-categorized as follows: cardiac/aortic aneurysm repair/abdominal open surgery, laparoscopic abdominal, neurosurgery, and other. To identify risk factors independently associated with the occurrence of PPCs, we conducted a multivariable analysis using a logistic regression model with backward step-wise selection with p<0.05 for inclusion of variables and p>0.10 for removal of variables. Variables were assessed for collinearity and goodness-of-fit of the model was evaluated with the Hosmer–Lemeshow test. Initial candidate variables for multivariable analysis were all of the variables we collected prospectively, regardless of p value in the univariate analyses.

**Table 1:** Baseline characteristics (n = 2,059).

	Mean ± SD or number (%)
Age, year	67±11
Sex, male	1288 (62.6)
Body mass index, kg/m^2^	23.7±3.5
Current smoker within 2 months	300 (14.6)
Alcoholics	77 (3.7)
Comorbidities	
Malignancy	844 (41.0)
CHF	85 (4.1)
General status	
Confused mentality	35 (1.7)
Recent loss of body weight	76 (3.7)
ASA physical status classification	1.7±0.6
1	749 (36.4)
2	1129 (54.8)
3	166 (8.1)
4	13 (0.6)
5	2 (0.1)
Pulmonary Function Test, Laboratory and radiographic findings	
Airflow limitation	613 (29.8)
Serum albumin, g/dl	4.1±0.5
Serum hemoglobin, g/dl	13.0±1.9
Active lesion on chest radiography	210 (10.2)
Type of anesthesia	
General	1769 (85.9)
Spinal	230 (11.2)
Combined spinal and epidural	46 (2.2)
Monitored anesthesia care	14 (0.7)
Emergency surgery	84 (4.1)
Surgical specialty	
Abdominal	643 (31.2)
Upper abdominal	254 (12.3)
Lower abdominal	158 (7.7)
Laparoscopic abdominal	231 (11.2)
Orthopedic and spinal	418 (20.3)
Neurosurgery	323 (15.7)
Gynecologic and urologic	294 (14.3)
Head and neck	120 (5.8)
Cardiac surgery	63 (3.1)
Vascular	56 (2.7)
Aortic aneurysm repair	29 (1.4)
Others*	113 (5.5)

*included 49 endocrine, 35 eye and plastic, and 29 breast surgery.

SD, standard deviation; CHF, congestive heart failure; ASA, American Society of Anesthesiologists.

From the multiple logistic regression model findings, a score-based predictive scoring system was developed. We assigned scores according to dividing beta coefficients by the absolute value of the smallest coefficient in the final model and rounding up to the nearest integer to generate a simple integer-based point score for each predictor variable [11]. The total score for each patient was calculated by adding each component together. Using a receiver operating characteristic (ROC) curve, we explored the predictive value of the newly developed scoring system for the occurrence of PPCs, and internally validated the rule using the bootstrap method in the original dataset by sampling with replacement for 1,000 iterations.

All tests were two-sided, and a p-value of less than 0.05 was considered to indicate statistical significance. Statistical analyses were performed using the SPSS software (IBM SPSS Statistics ver. 21, Chicago, IL) and STATA 12 (STATA, College, Texas, USA) for ROC analysis.

## Results

### Patient characteristics

**Figure 1:**
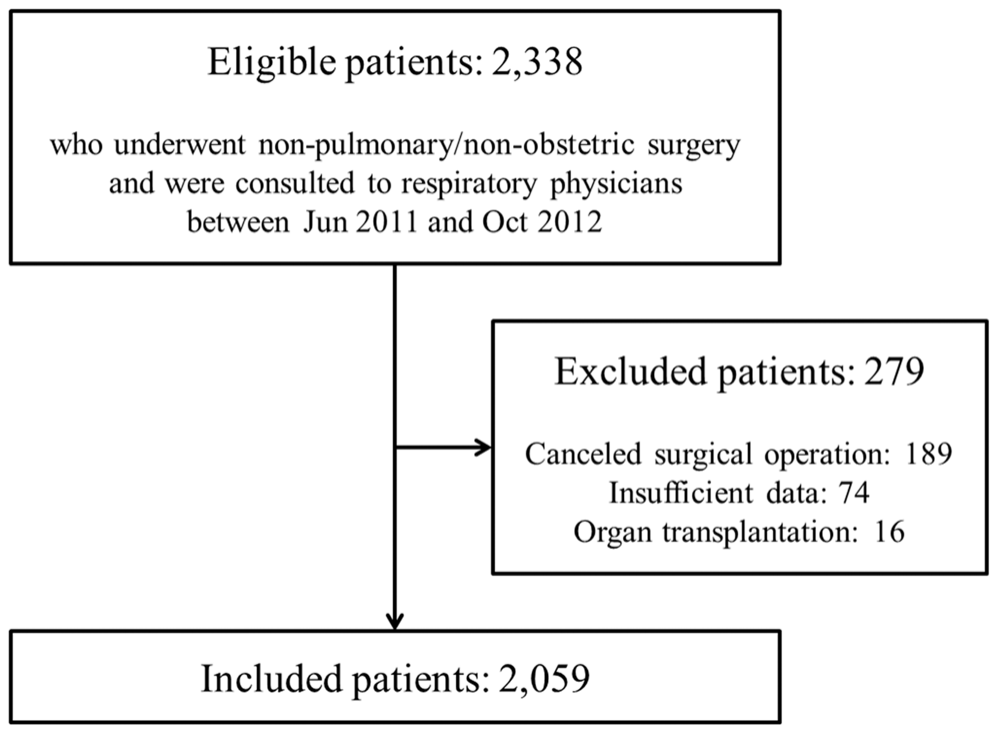
Recruitment flow chart.

During the study period, 2,338 consecutive patients had respirology consultations to evaluate the risk of PPCs (Fig. 1). Of these, 189 patients whose surgery was canceled, 16 who underwent organ transplantation, and 74 who had a consultation with a respiratory physician but variables were not registered were excluded. Consequently, 2,059 patients were eligible for this study.

Their baseline characteristics are presented in Table 1. The mean age of the patients was 67 years and 62.6% were males. In total, 844 (41.0%) patients had malignancies and 85 (4.1%) had CHF. Most patients were classified in ASA classes 1 (36.4%) and 2 (54.8%) and 613 (29.8%) had airflow limitation. The most frequent types of surgery were abdominal surgery (31.2%) and orthopedic and spinal surgery (20.3%). Of the 643 abdominal surgeries, 231 (35.9%) patients underwent laparoscopic surgery.

### Clinical outcome

The prevalence and number of PPCs according to each type of surgery is presented in Table 2. In total, 183 PPCs were recorded in 140 (6.8%) patients. The most common PPCs were respiratory failure (3.4%), pleural effusion (1.8%), atelectasis (1.7%), and respiratory infection (1.6%). The mean duration of anesthesia was 3.5±2.0 h and the median length of hospital stay was 10 (6–15) days in all patients.

**Table 2:** Prevalence of postoperative pulmonary complications according to each type of surgery.

	Patients	Respiratory failure	Pleural effusion	Atelectasis	Pneumonia	Bronchospasm	Pneumothorax	Number of occurred PPCs	Number of patients who occurred PPCs
Abdominal*	643	36	29	27	17	1	0	110	82
Upper abdominal	254	16	21	15	6	0	0	58	48
Lower abdominal	158	15	7	6	7	1	0	36	22
Laparoscopic abdominal	231	5	1	6	4	0	0	16	12
Orthopedic and spinal	418	15	1	1	8	2	0	27	23
Neurosurgery	323	4	1	0	5	0	0	10	7
Gynecologic and urologic	294	4	1	0	2	0	0	7	5
Head and neck	120	0	0	0	0	0	0	0	0
Cardiac	63	4	2	1	1	1	1	10	9
Vascular	56	3	1	0	0	0	0	4	4
Aortic aneurysm repair	29	5	3	6	0	1	0	15	10
Others	113	0	0	0	0	0	0	0	0
Total	2059	71	38	35	33	5	1	183	140

*included upper abdominal, lower abdominal, and laparoscopic abdominal surgery.

PPC, postoperative pulmonary complications.

Cases are duplicated.

### Predictors of PPC and the new scoring system

A multivariable logistic regression model for evaluating risk factors of the occurrence of PPCs is shown in Table 3. The multivariable logistic regression model selected seven independent predictors of PPCs: age ≥70 years (odds ratio (OR) 2.03; 95% confidence interval (CI) 1.37–3.00; p<0.001), current smoker within 2 months (OR 1.62; 95% CI 1.03–2.54; p = 0.036), ASA class ≥2 (OR 1.59; 95% CI 1.01–2.52; p = 0.046), airflow limitation (OR 1.75; 95% CI 1.18–2.58; p = 0.005), serum albumin <4.0 g/dL (OR 1.49; 95% CI 1.00–2.21; p = 0.049), emergency surgery (OR 2.16; 95% CI 1.00–4.65; p = 0.050), and cardiac/aortic aneurysm repair/abdominal open surgery (OR 7.02; 95% CI 4.04–12.19; p<0.001).

**Table 3:** Results of logistic regression model to predict postoperative pulmonary complications (PPC) and assigned points based on regression coefficient to develop the PPC scoring index.

	Univariable analysis				Multivariable analysis			
Variables	With PPC (n = 140)	Without PPC (n = 1919)	OR (95% CI)	p	Regression coefficient	OR (95% CI)	p	Assigned points
Age ≥70 yr	82 (59)	822 (43)	1.89 (1.33–2.67)	<0.001	0.708	2.03 (1.37–3.00)	<0.001	2
Sex, male	102 (73)	1186 (62)	1.66 (1.13–2.44)	0.010				
Body mass index								
<18.5 kg/m^2^	12 (9)	114 (6)	1.40 (0.68–2.89)	0.594				
18.5–25.0 kg/m^2^	87 (62)	1158 (60)	1.00	Ref				
>25.0 kg/m^2^	41 (29)	647 (34)	0.84 (0.54–1.31)	0.768				
Current smoker within 2 months	33 (24)	267 (14)	1.91 (1.27–2.88)	0.002	0.482	1.62 (1.03–2.54)	0.036	1
Alcoholics	4 (3)	73 (4)	0.75 (0.27–2.08)	0.580				
Malignancy	78 (56)	766 (40)	1.89 (1.34–2.68)	<0.001				
CHF	7 (5)	78 (4)	1.24 (0.56–2.75)	0.592				
Confused mentality	2 (1)	33 (2)	0.83 (0.20–3.49)	0.797				
Recent loss of body weight	11 (8)	65 (3)	2.44 (1.26–4.73)	0.009				
ASA physical status ≥ class 2	109 (78)	1201 (63)	2.10 (1.40–3.17)	<0.001	0.466	1.59 (1.01–2.52)	0.046	1
Presence of airflow limitation	68 (49)	545 (28)	2.38 (1.69–3.37)	<0.001	0.558	1.75 (1.18–2.58)	0.005	1
Serum albumin <4.0 g/dl	61 (44)	547 (29)	1.95 (1.37–2.77)	<0.001	0.398	1.49 (1.00–2.21)	0.049	1
Serum hemoglobin <10 g/dl	16 (11)	129 (7)	1.79 (1.03–3.10)	0.039				
Active lesion on chest radiography	18 (13)	192 (10)	1.33 (0.79–2.22)	0.284				
General anesthesia	129 (92)	1640 (85)	2.00 (1.06–3.74)	0.031				
Emergency surgery	10 (7)	74 (4)	1.92 (0.97–3.80)	0.062	0.768	2.16 (1.00–4.65)	0.050	2
Surgical specialty								
Others	32 (23)	969 (50)	1.00	Ref		1.00		
Cardiac/aortic aneurysm repair/abdominal open surgery	89 (64)	415 (22)	6.49 (3.89–10.85)	<0.001	1.949	7.02 (4.04–12.19)	<0.001	4
Laparoscopic abdominal	12 (9)	219 (11)	1.66 (0.72–3.81)	0.432	0.551	1.74 (0.74–4.09)	0.369	
Neurosurgery	7 (5)	316 (16)	0.67 (0.24–1.84)	1.000	−0.268	0.77 (0.25–2.30)	1.000	

A multivariable analysis using a logistic regression model was conducted with backward step-wise selection with p<0.05 for inclusion of variables and p>0.10 for removal of variables from all variables used for Univariable analysis and only significant variables in a multivariable analysis were indicated in the table.

PPC, postoperative pulmonary complications; OR, odds ratio; CI, confidence interval; CHF, congestive heart failure; ASA, American Society of Anesthesiologists.

Hosmer-Lemeshow test, χ^2^ = 7.653, p = 0.468.

Of these seven predictors, the lowest logistic coefficient was 0.398 for serum albumin <4.0 g/dL. On the basis of this value, we developed a new scoring system to predict the occurrence of PPCs, composed of age ≥70 years (2 points), current smoker within 2 months (1 point), ASA class ≥2 (1 point), the presence of airflow limitation (1 point), serum albumin <4.0 g/dL (1 point), emergency surgery (2 points), and cardiac/aortic aneurysm repair/abdominal open surgery (non-laparoscopic) surgery (4 points), resulting in a maximum of 12 possible points. The new predictive scoring system had a high discriminatory power to predict the occurrence of PPCs with an area under the curve (AUC) of 0.79 (95% CI 0.75–0.83), which was similar to 0.79 (95% CI 0.75–0.82) for the average AUC obtained from an internal validation. Moreover, the AUC of this scoring system was significantly higher than the AUC of 0.65 (0.61–0.69) from predictive scoring summed simply as 1 point for each variable (maximum of 7 possible points) (p<0.001).

**Figure 2:**
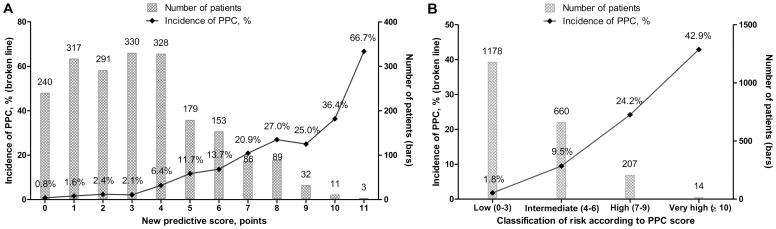
The number of patients and the incidence of postoperative pulmonary complications (PPCs) by each point of the PPC scoring index (A) and by each risk class according to the PPC score (B). PPC scores were calculated as follows: 1 point each for current smoker within 2 months, the presence of airflow limitation, American Society of Anesthesiologists ≥ class 2, and serum albumin <4.0 g/dL; 2 points each for age ≥70 years and emergency operation; and 4 points for cardiac/aortic aneurysm repair/abdominal open surgery without a laparoscopic procedure. PPC risk was classified according to the PPC score as follows: low, 0–3 points; intermediate 4–6 points; high, 7–9 points; very high, ≥10 points. Each bar graph shows the number of patients with each score for the PPC scoring index. Each broken line shows the incidence of PPCs for each point on the PPC scoring index. PPCs, postoperative pulmonary complications.

Figure 2 shows the incidence of PPCs and the number of patients by each point (Fig. 2A) and each class (Fig. 2B) of the scoring system. Each point increment was associated with a 1.6-fold (OR 1.56; 95% CI 1.45–1.68, p<0.001) increase in the odds for the occurrence of PPCs. When patients were grouped into low- (0–3 points), intermediate- (4–6 points), high- (7–9 points), and very high-risk (≥10 points) strata as a function of the overall point score, the occurrence of PPCs was associated with a 3.8-fold (OR 3.80; 95% CI 3.04–4.77; p<0.001) increment by each class increment.

## Discussion

This study evaluated risk factors for the occurrence of PPCs among more than 2,000 patients who underwent surgery with general or neuraxial anesthesia and the overall incidence of PPCs was 6.8%. Using multivariable logistic regression models, we found seven independent predictors of PPCs: age ≥70 years, current smoker within 2 months, ASA class ≥2, the presence of airflow limitation, serum albumin <4.0 g/dL, emergency surgery, and cardiac/aortic aneurysm repair/abdominal open surgery. When each independent variable was endowed with a weighted score based on logistic coefficients, cardiac/aortic aneurysm repair/abdominal open surgery was the most predictive variable with 4 points, followed by age ≥70 years and emergency surgery with 2 points each. Compared with the predictive scoring system based on simply scoring 1 point for each variable, this weighted risk score strategy had a significantly better discriminative power for estimating PPCs.

Using the variables listed in the ACP guidelines, we derived new cut-off values for each variable using our cohort. The newly determined cut-off values from our cohort were 70 years of age, class 2 of ASA physical status, and 4.0 mg/dL for albumin. While advanced age and limited physical status were similar to the findings of previous studies, the cut-off value for albumin was considerably higher than that suggested in previous studies (albumin <3.0–3.5 mg/dL) [5], [12]. Overall, the percentage of patients with albumin less than 3.0 g/dl was only 4.2%, which reflects the fact that the patients who underwent surgery had good nutritional status.

The incisions for chest and abdominal operations frequently damage the diaphragm and respiratory muscles, and postoperative pain provokes hypoventilation [13]–[15]. Compared to open surgery, the laparoscopic approach for abdominal surgery causes less postoperative pain, facilitating deep breathing and active coughing to prevent PPCs. Previous studies have shown that open abdominal surgery had a nearly twofold increased risk of postoperative complications compared to laparoscopic abdominal surgery [16], [17] and laparoscopic abdominal surgery offers improved postoperative lung function [18]. As such, for the first time, we evaluated laparoscopic abdominal surgery as a separate variable for PPCs. Our data showed that open abdominal surgery was an independent risk factor for PPCs, while laparoscopic abdominal surgery was not; confirming that opting for laparoscopic procedures is an important strategy to reduce the rate of PPCs.

Contrary to previous studies [19]–[21], we did not identify CHF as a significant risk factor for PPCs. As two of the three previous papers investigated only geriatric patients (age 70 and older), a history of CHF might be a major risk factor for selected patients of advanced age (≥70 years) [20], [21]. A recent study, which had patents with median age of 60 years old and used a similar definition of PPCs to our study, also showed that CHF was not an independent risk factor [7]. However, since the patients with severe heart failure might be referred to cardiologists only, and not to respiratory physicians, selection bias might affect our results. Regarding obesity, our study was consistent with most reports that increased BMI was not a major factor for PPCs [10], [22], [23].

In our study, all variables were registered prospectively by specialists in respiratory medicine after examining the patients. In addition, all patients scheduled for surgery were advised to undergo perioperative strategies to prevent PPCs. Particularly, as incentive spirometry has been shown to correlate with a reduction in the incidence of respiratory infection, respiratory failure, and atelectasis and with lung function improvement [6], [24], it has been recommended that incentive spirometry be used routinely during the perioperative period in all patients undergoing general anesthesia.

There were several limitations to this study. First, there was its retrospective nature for evaluating the main outcome of PPCs. Second, there were 38,225 cases with non-pulmonary/non obstetric surgical operations during the period of this study. Since the consultations with respiratory physicians were at the discretion of the treating surgeons, 2,059 patients (5.4%) were included in our cohort. Thus, selection bias might have influenced our findings despite the use of prospectively registered data. Given that more than 75% of cohort patients were older than age of 60 years and 30% had the presence of airflow limitation, our cohort would reflect real world practice. Third, this study was from a single institution, a tertiary referral center, which might limit the generalizability of our findings. Finally, our scheme needs to be validated by prospective studies.

In conclusion, a new risk stratification system that comprises seven factors with weighted scores had good discriminative ability for predicting PPCs in our study cohort. Our findings raise the possibility of using new cut-off values for albumin and inclusion of laparoscopic procedures as a separate variable. Additional studies are required to validate this new prediction rule.

## Supporting Information

Table S1 **Cut points of the continuous variables in the logistic regression.**
(DOCX)
