# Supplementary material for: Development of a Prediction Rule for Estimating Postoperative Pulmonary Complications
Source: PLoS One. 2014 Dec 1;9(12):e113656. doi: 10.1371/journal.pone.0113656 (PMC4249954; doi:10.1371/journal.pone.0113656)
Supplement: Table S1 — Cut points of the continuous variables in the logistic regression. (DOCX) [file pone.0113656.s001.docx]

**Development of a prediction rule for estimating postoperative pulmonary complications**

Byeong-Ho Jeong^1^*, Beomsu Shin^1^*, Jung Seop Eom^2^, Hongseok Yoo^1^, Wonjun Song^1^, Sangbin Han^3^, Kyung Jong Lee^1^, Kyeongman Jeon^1^, Sang-Won Um^1^, Won-Jung Koh ^1^, Gee Young Suh^1^, Man Pyo Chung^1^, Hojoong Kim^1^, O Jung Kwon^1^, Sookyoung Woo^4^, Hye Yun Park^1^

^1^Division of Pulmonary and Critical Care Medicine, Department of Medicine, Samsung Medical Center, Sungkyunkwan University School of Medicine, 81 Irwon-ro, Gangnam-gu, Seoul, Republic of Korea.

^2^Department of Internal Medicine, Pusan National University College of Medicine, Pusan, Republic of Korea.

^3^Department of Anesthesiology and Pain Medicine, Samsung Medical Center, Sungkyunkwan University School of Medicine, 81 Irwon-ro, Gangnam-gu, Seoul, Republic of Korea.

^4^Biostatistics Team, Samsung Biomedical Research Institute, 81 Irwon-ro, Gangnam-gu, Seoul, Republic of Korea.

* These authors contributed equally to this work.

**Corresponding author:** Hye Yun Park M.D.

Division of Pulmonary and Critical Care Medicine, Department of Medicine, Samsung Medical Center, Sungkyunkwan University School of Medicine, 81 Irwon-ro, Gangnam-gu, Seoul, Korea. 135-710

**Table S1** Cut points of the continuous variables in the logistic regression.

| **Variables** | **Cut points** | **Sensitivity** | **Specificity** | **PPV** | **NPV** | **Youden’s index** |
| --- | --- | --- | --- | --- | --- | --- |
| Age | ≥ 70 yr | 58.6 (44.9–66.8) | 57.2 (54.9–59.4) | 9.1 (7.3–11.1) | 95.0 (93.6–96.2) | 15.8 |
| Serum albumin | < 4.0 mg/dl | 44.2 (35.8–52.9) | 71.1 (69.0–73.1) | 10.0 (7.8–12.7) | 94.6 (93.3–95.7) | 15.3 |
| ASA physical status | ≥ class 2 | 77.9 (70.1–84.4) | 37.4 (35.2–39.6) | 8.3 (6.9–9.9) | 95.9 (94.2–97.2) | 15.3 |

PPV, positive predictive value; NPV, negative predictive value; ASA, American Society of Anesthesiologists.
